# Variation in Soil Organic Carbon under Different Forest Types in Shivapuri Nagarjun National Park, Nepal

**DOI:** 10.1155/2021/1382687

**Published:** 2021-11-03

**Authors:** Jun Shapkota, Gandhiv Kafle

**Affiliations:** Faculty of Forestry, Agriculture and Forestry University, Hetauda, Nepal

## Abstract

Understanding distribution of soil organic carbon (SOC) in soil profile is important for assessing soil fertility and SOC stock because it varies with soils of different vegetation and land use types. In this context, the objective of this research is that it was conducted to determine key variance in the SOC stock in three different soil layers, 0–20 cm, 20–40 cm, and 40–60 cm of different vegetation covers of Shivapuri Nagarjun National Park of Kathmandu district, Nepal. Overall field measurement was based on standard national methods. We used the dichromate digestion method to analyse SOC concentrations. The highest SOC concentration (%) was recorded as 4.87% in 0–20 cm of oak forest and lowest 0.42% in 40–60 cm of Chir pine forest. Forest types (oak, upper mixed hardwood, lower mixed hardwood, and Chir pine) had SOC stock 149.62, 104.47, 62.5, and 50.85 t/ha, respectively, up to 60 cm depth. However, these values are significantly different (*p*=0.02) at 5% level of significance when comparing means between the forest types. The SOC stock was decreased with increased soil depth, though not significantly different at 5% level of significance. Further study with respect to different climate, soil, forest, and land use type is recommended.

## 1. Introduction

Soil is a complex mixture of mineral nutrients, organic matter, water, air, and living organisms determined by diverse environmental factors such as climate, parent material, relief, organisms, and time factor [1]. Soil is also the substratum of all living organisms in the earth's ecosystems, which contains nutrient reserves and enables many biological processes for vegetation development. Soil is the greatest carbon pool in terrestrial biosphere [2]. The aboveground input obtained from organic biomass and root inputs stores carbon in the soil [3].

Soil organic matter (SOM) is an essential source of plant nutrients that affects the rate of organic residues and inorganic fertilizers and increases soil accumulation, which can reduce soil degradation, and also increases the exchange of cations and retaining capacity of water. Soil organic carbon (SOC) stock is an important part of global carbon cycle involving the cycling of carbon through the soil, plants, ocean, and atmosphere. In the first meter of soil, the SOC stock contains an estimated 1500 PgC which is more carbon than in the atmosphere (approximately 800 PgC) and terrestrial plants (500 PgC) combined [4]. SOC stock has gained worldwide attention in recent years in the context of the international CO_2_ emission policy agendas [5]. The amount of SOC stock experiences significant spatial diversity, both horizontally by land use type and vertically within the soil profile. Depending on their turnover times, land cover affects carbon that exists between different soil pools. Soils are a potentially viable sink for atmospheric carbon [6].

Forest plays key role in the global carbon cycle, as it sequesters a significant amount of carbon stock from the atmosphere. The accumulation of carbon in forests can be both carbon sources and sinks, depending on the particular management regime and activities [7]. Biotic influences, such as the abundance and vigor of faunal, microbial, and plant species, as well as environmental factors temperature, moisture, etc., influence the stability and distribution of SOC in the soil profile [8]. Factors that control the decomposition of SOM in soil include soil temperature and water content (mainly determined by climatic conditions) which greatly influence storage of SOC through its effect on microbial activity [4]. Factors that may be important for increasing SOC storage include litter production (both above and below ground); litter quality; placing organic matter deeper in the soil either directly by increasing below-ground inputs or indirectly by improving surface mixing by soil organisms; and increasing physical protection through either intra-aggregate or organic mineral complexes and microclimate change. The protection of SOC stocks in forest soils to increase carbon sequestration is crucial in maintenance of carbon balance. Das and Mondal [9] found that litter production was continuous, but the quantity of litter produced varied by season and dry winter period showed maximum litterfall of the studied species at Ramna forest. Nutrients of N, K, and P were the primary limiting nutrients returned to soil through litterfall with important roles in soil fertility and forest productivity of *Shorea robusta* and *Tectona grandis* in a subtropical forest of West Bengal, Eastern India. Morales-Ruiz et al. [10] found that fine root production correlated positively with SOC concentration in tropical silvopastoral systems. The results presented by Chen et al. [11] showed that favorable climate conditions, particularly high precipitation, tend to increase both species richness and below-ground biomass, which had a consistent positive effect on SOC stock in forests, shrublands, and grasslands.

Rapid change in land use and land cover in Nepal has been experienced due to the high population growth. Nepal is facing serious problems of depletion of soil quality and resultant low crop yields due to numerous human activities and the change in land use resulting in decline of SOM, nutrients, and their hydrological parameters [12]. Land use and vegetation type affects soil erosion and SOC dynamics by its effect on SOC stock, CO_2_ flux, and soil leaching from dissolved organic carbon (DOC). Soil, thus, may be an atmospheric carbon source or sink depending on land use and soil management.

A joint five-year study undertaken by the Department of Forest Research and Survey and the National Forest Products Survey Project reveals 44.7% of Nepal's total area of 147,181 square kilometers. Out of this, 40.36% (5.9 m ha) are forests and the remaining 4.38% (0.6 m ha) are scrublands and the other is forest land. The average SOC stock in the forest of Nepal was estimated as 176.9 t/ha. Of the overall forest SOC stock, 61.5%, 37.8%, and 0.6% were tree components (live, dead standing, dead wood and underground biomass), forest soils, and litter and debris, respectively [13].

Nepal's Department of Forest Research and Survey (DFRS) reported in 2015 that Nepal had an average organic carbon content of 66.8 t/ha, 1.1 t/ha, and 108.8 t/ha in soil, litter and debris, and tree components (more than 10 cm diameter at breast height). In high mountain and high himal areas, the largest SOC stock (114.0 t/ha) has been estimated. With an average of 31.4 t/ha, SOC stock was the lowest in the Churia region. Middle mountain area showed an average SOC stock of 54.3 t/ha. SOC stocks in the Terai forests were found to be slightly higher than those in Churia.

Although most SOC researches have so far focused on surface layers (generally as low as 30 cm), it is also increasingly recognized that subsurface soils play an important role in SOC storage, especially given the higher total volumes and bulk densities of these soils and the greater stability and durability of SOC than in surface soils [14]. With increased soil depths, SOC and nitrogen were found to decrease, with a statistically significant difference in values across various soil layers. The bulk density and C/N ratio of the soil increased as the soil depths increased [15–17]. In this context, this research was conducted to determine key variance in the SOC stocks in different forest types and soil depths of Shivapuri Nagarjun National Park of Kathmandu district, Nepal.

## 2. Materials and Methods

### 2.1. Research Site

**Figure 1:**
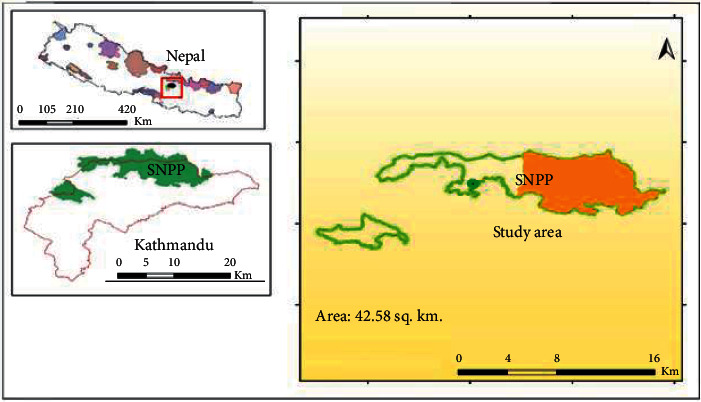
Study area map.

This study is carried out in Shivapuri Nagarjun National Park (SNNP) which lies in the midhills of Kathmandu district, Nepal (Figure 1). Generally, the forest of Shivapuri lies between 27°45′ and 27°52′ N latitude and 85°16′ and 85°45′ E longitude, while the forest of Nagarjun lies between 27°43′ and 27°46′ N latitude and between 85°13′ and 85°18′ E longitude with the elevation range from 1350 to 2732 m covering a total area of 159 km^2^. The annual temperature of the district varies from 19°C to 30°C during autumn and from 2°C to 17°C during winter, whereas the climate varies from subtropical to temperate. The annual precipitation is of about 1,400 mm which falls mostly from May to September, with 80% during monsoon. The area has high floral diversity due to its altitudinal, location, and climatic variations. A total of 1250 species of vascular plants and 129 species of mushrooms have been recorded in the park. *Schima-Castanopsis, Pinus,* and *Quercus*-*Rhododendron* are the dominant tree species of the park. SNNP has four major forest types as follows:

- Lower mixed hardwood forests at 1000–1500 m,
- Chir pine forests at 1000–1600 m,
- Oak forests at 2300–2700 m, and
- Upper mixed hardwood forests at 1500–2700 m.

**Figure 2:**
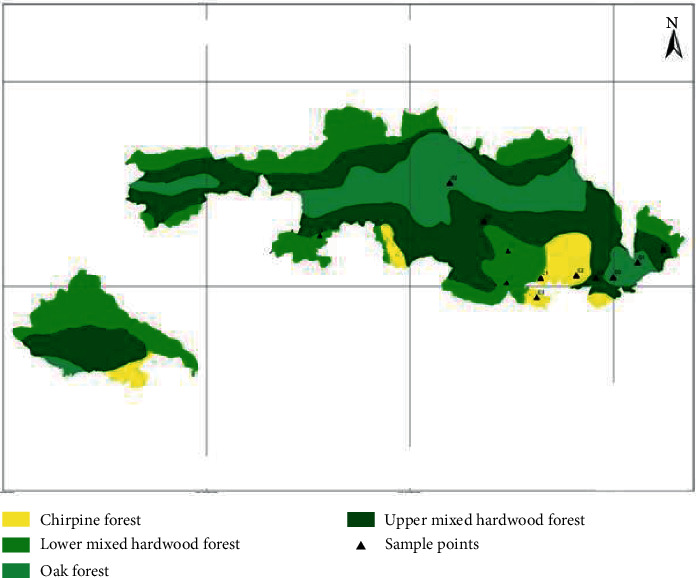
Vegetation map showing forest types of SNNP and sample points.

The common tree species are *Schima wallichii, Pinus roxburghii, Castanopsis indica, Alnus nepalensis, Myrica esculenta, Quercus semecarpifolia, Rhododendron arboretum, Juglans regia, Taxus wallichiana*, etc. The vegetation map of the SNNP is presented in Figure 2.

### 2.2. Soil Sampling

Stratified random sampling method was applied to layout plots in the study area from four forest types to estimate profile storage of SOC stock. A total of 12 plots were selected randomly, three from one forest. Three replicates of soil pit up to 60 cm depth from each forest types were dug for soil sample collection. Undisturbed soil samples, each around 800 grams, from the 0–20 cm, 20–40 cm, and 40–60 cm soil depths were taken by a cylindrical core sampler (5.7 cm diameter and 20 cm height) and were packed in plastic bags, labelled, and taken to the laboratory of National Environmental and Scientific Services (NESS) Pvt., Ltd., Kathmandu.

### 2.3. Soil Analysis

Soil bulk density was determined using the soil core samples [18]. Soil sample was transported to the laboratory for oven drying and measuring the oven dry weight after drying 24 hours at constant temperature of 105°C.

(1) $$\begin{array}{c} \text{Bulk density of soil}=\frac{\left(\text{oven dry weight of soil in gram}\right)}{\left({\text{volume of the soil in cm}}^{3}\right)}. \end{array}$$

For the soil sample containing stone, volume of the soil (*v*) was determined by

*v* = volume of core−volume of stone (determined by displacing water).

SOC concentration was analyzed using Walkley–Black wet oxidation method [19, 20]. This method involves oxidation of organic matter by potassium dichromate with sulphuric acid followed by titration. Samples from each of the three horizons were prepared for carbon measurement by removing stones and plant residue >2 mm as well as by grinding. Total SOC stock was calculated by using the given formula by De Vos et al. [21].

SOC stock (t/ha) = SOC concentration (%) × soil bulk density (gm/cm^3^) × soil layer depth (cm).

The significant difference of bulk density and SOC stock was tested using ANOVA at 5% level of significance and the correlation test was applied to find out relationship between the variables. MS Excel was used for statistical analysis of the data.

## 3. Results

### 3.1. Soil Bulk Density

The soil bulk density (BD) increased with soil depths for all forests. The minimum BD, 0.6 gm/cm^3^, was found at 0–20 cm depth in the oak forest and the maximum BD, 0.9gm/cm^3^, was found at 40–60 cm depth in Chir pine forest (Table 1).

SOC concentration was found from 0.4% to 4.8% in the study area (Table 2).

The SOC concentration was found higher in the upper layers which could be related to higher soil organic matter content and less influence of parent materials. The oak forest soil had the highest SOC concentration (4.8%), followed by upper mixed hardwood forest (3.7%), lower mixed hardwood forest (1.9%), and Chir pine forest (1.5%). In almost all cases, SOC concentration has been found higher at the upper level of soil. Pradhan et al. [22] reported greater SOC concentration in the *Schima-Castanopsis* forest than in other forest types, but this study shows that the SOC concentration is found higher in the oak forest as the forest was dense and due to different microbial activities in the soil. The greater the depth, the lower the SOC concentration for all types of forest.

### 3.2. Total SOC Stock

The total SOC stock followed the order as oak forest > upper mixed hardwood > lower mixed hardwood > Chir pine forest with the total mean SOC stocks in each forest types in soil profile up to 60 cm depth being 149.6, 104.4, 62.5, and 50.8 t/ha, respectively (Table 3).

### 3.3. Comparison of SOC Stock between Forests

ANOVA test for comparison of variance in total SOC stock between vegetation types showed that there is significant difference with *p* value 0.021 (Table 4). Similarly, there was significant difference in BD and SOC concentration also at 5% level of significance with *p* values 0.014 and 0.019, respectively, for which alternative hypothesis is accepted, being *p* value less than 0.05 (Table 5). There was significant difference in SOC stocks in different forest types (Table 6).

### 3.4. ANOVA Test for Comparison of SOC between Soil Layers

ANOVA test for comparison of mean SOC stock between soil layers from four types of forests showed that there is significant difference in Chir pine forest (*p*=0.003) (Table 7) and no difference was observed in lower mixed hardwood (*p*=0.47), upper mixed hardwood (*p*=0.55), and oak forest (*p*=0.13) at 5% level of significance (Tables 8, 9, and 10).

### 3.5. Relation between BD, SOC Concentration, and Total SOC Stock

**Figure 3:**
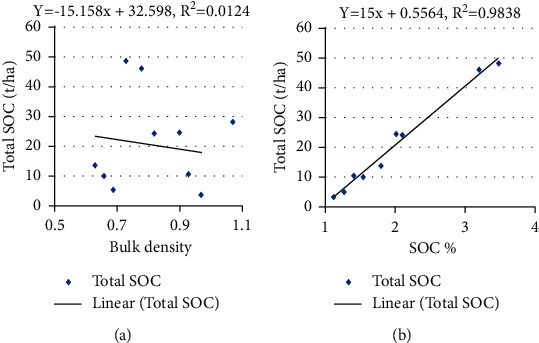
(a) SOC concentration (t ha^−1^) and BD. (b) SOC stocks (t ha ^−1^) with SOC concentration in lower mixed hardwood forest.

**Figure 4:**
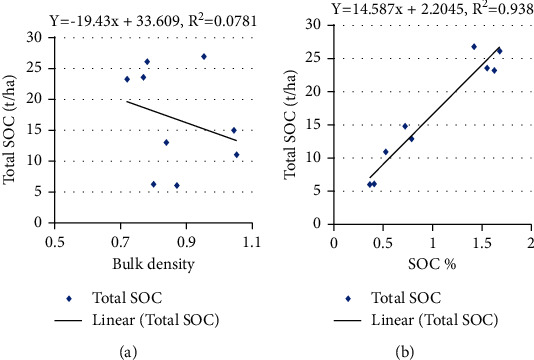
(a) SOC stocks (t/ha) and BD. (b) SOC stock (t/ha) with SOC concentration in Chir pine forest.

**Figure 5:**
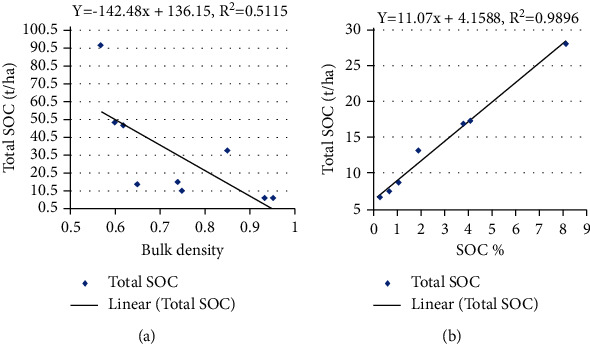
(a) SOC stocks (t/ha) and BD. (b) SOC stock (t/ha) with SOC concentration in upper mixed hardwood forest.

**Figure 6:**
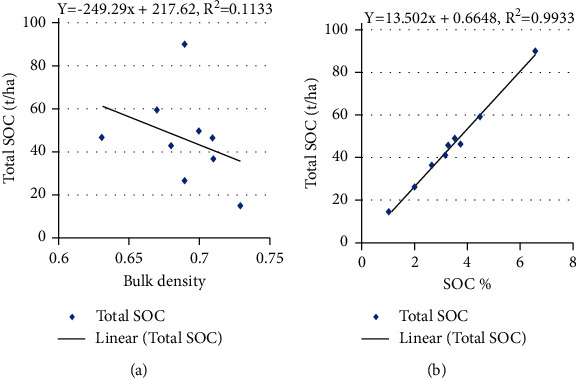
(a) SOC stocks (t/ha) and BD. (b) SOC stock (t/ha) with SOC concentration in oak forest.

The BD depends on several factors such as compaction, consolidation, and SOC stock present in the soil, but it is negatively correlated to the organic carbon content, and as the organic matter increases, the bulk density of soil decreases which is required for the proper growth of the plants [23]. This study shows that there was no linear relation between BD and SOC concentration with adjusted *R*^2^ values 0.01, 0.07, 0.51, and 0.11 for lower mixed hardwood, Chir pine, upper mixed hardwood, and oak forest (Figures 3–6). Chaudhari et al. [23] also showed that there was high degree reverse correlation between SOM and BD of soil. However, there was linear relationship between total SOC stock and SOC concentration with adjusted *R*^2^ values 0.98, 0.93, 0.98, and 0.99 for lower mixed hardwood, Chir pine, upper mixed hardwood, and oak forest. So, the SOC stock is governed by the bulk density and SOC concentration of the soil. Similar result was also obtained from the study of Ghimire et al. [24].

## 4. Discussion

A combination of factors, particularly climate, parent material (or soil type), and vegetation cover or land management, is required to understand and make meaningful estimates of levels of SOC stock [14]. There was a gradual increase of BD with the increase in soil depth for all four forest types. The top soil layer had lower BD indicating that the soil was better for plant growth compared to other soil depths which could be attributed to the higher SOC concentration in the top layer of soil. However, the BD was found to have decreased from top to bottom with the increase in the soil depth in different ecological regions [16, 25]. Therefore, the change trend of SOC stock among the different vegetation types was mainly determined by the variation in BD values and SOC concentration.

Higher BD was found in Chir pine forest which was probably due to compaction of soil. A lower turnover of SOM in soil often leads to soil compaction which increases BD and decreases pore volume because the soil found in Chir pine forest was mostly sand formed due to collusion of rock and seemed to minimize SOM in the soil. Due to lower SOM content, less aggregation, fewer roots and other soil dwelling organisms, and soil compaction, the BD of soil will increase with increasing soil depth. Pradhan et al. [22] reported that the organic matter content of soil decreases with increasing soil depth and that this results in a decrease in soil porosity and also in soil compaction. With the increase in depth in each forest type, a gradual increase in bulk density was seen. The bulk density increases as we go deeper and deeper because of the natural soil compaction. The oak forest soil had the highest SOC concentration (4.8%), followed by upper mixed hardwood forest (3.7%), lower mixed hardwood forest (1.9%), and Chir pine forest (1.5%). In almost all cases, SOC content has been found higher at the upper level of soil. Pradhan et al. [22] also reported greater SOC concentration in the *Schima-Castanopsis* forest than in other forest types, but this study shows that the SOC concentration is found higher in the oak forest as the forest was dense and due to different microbial activities in the soil. The greater the depth, the lower the SOC concentration for all types of forest. ANOVA test showed that there was significant difference in mean SOC stock with *p* value 0.02 between vegetation types at 5% level of significance. But there was no significant difference between soil layers in oak (*p*=0.13) and lower mixed (*p*=0.47) and upper mixed forests (*p*=0.55), but it showed significant difference in Chir pine forest (*p*=0.003). The higher SOC concentration in the top layer may be due to rapid decomposition of forest litter. Soils with rich levels of SOC stock generally indicate high fertility, and therefore, it is important to maintain its optimum level that requires a careful land use and management practices [15].

However, many more studies reported that focusing on subsoil would give an accurate estimation of changes in SOC concentration and stock. This study showed that the SOC stock at 0–60 cm depth in the lower mixed hardwood, Chir pine, upper mixed hardwood, and oak forests accounted for 62.5 t/ha, 50.8 t/ha, 104.7 t/ha, and 149.6 t/ha, respectively.

Average SOC stock in our research (367.1 t/ha) was higher than the findings of Dahal [26] in pine forest (245.9 t/ha) and broad-leaved forest (163.9 t/ha) in midhills of Central Nepal [26] in pine forest (126.5 t/ha) and broad-leaved forest (49.7 t/ha) of midhills of Central Nepal; in a community forest (152.04 t/ha) in Kathmandu but was lower than [27] as found temperate forest of SNNP (599.5 t/ha). The higher value in the present study might be due to strict protection of mature forest and also due to difference in vegetation and topography. Litter decomposition was significantly slower in mature forests compared with secondary forests [28].

The SOC stock of oak forest was found to be higher than other forests as the forest was dense and due to lesser organic matter accumulation in the uppermost layer of the soil. The SOC and BD between three layers were found significantly different. SOC stock in the upper soil layer (0–20 cm) was found higher than the lower soil horizon (40–60 cm). This might be due to the variation in the time period of soil formation. The newly formed upper horizon could have contained more carbon. The nutrient could have been continuously restocked by SOM decomposition in the upper horizon, whereas the tree roots ingest more nutrients from the lower horizon. Nutrient leaching from the upper horizon is the source of lower horizon accumulation. Therefore, SOC stock is lesser in the lower horizon compared to the upper one. The higher nutrient accumulation in the upper horizon may have made the soil more porous, resulting in low BD. Similar result was reported by Mishra in SOC stock of Chapako Community Forest, Kathmandu, and Ranjitkar [27] in temperate forest of SNNP.

## 5. Conclusion

Forest types and soil depth both affected SOC stocks significantly. The highest SOC stock was observed in oak forest following the order of upper mixed hardwood forest, lower mixed hardwood forest, and Chir pine forest (149.6, 104.4, 62.5, and 50.8 t/ha), respectively. The effect of soil depth on SOC stock, BD, and SOC content was dependent on forest types. There is a declining trend of SOC stocks with increasing soil depths; however, the variation is not statistically significant. Bulk density (BD) was found to be increasing with increases in the depth of soil profile for all the land uses which shows negative co-relation with SOC. Overall, organic carbon stock in all forest types was seen lower as depth increased. This implies that the organic content of the soil is controlled by the bulk density and organic carbon percentage of the soil.

## Figures and Tables

**Table 1:** Bulk density (gm/cm^3^) of soil at different depths in different forest types.

Soil depth (cm)	Bulk density								Remarks
	Lower mixed hardwood (1000–1500 m)		Chir pine (1000–1600 m)		Upper mixed hardwood (1500–2700 m)		Oak (2300–2700 m)		
	Mean	SD	Mean	SD	Mean	SD	Mean	SD	Average
0–20	0.7	0.1	0.8	0.1	0.6	0.1	0.6	0.0	0.7
20–40	0.7	0.1	0.8	0.1	0.7	0.1	0.6	0.0	0.7
40–60	0.8	0.1	0.9	0.1	0.7	0.1	0.7	0.0	0.8
Average	0.7		0.8		0.7		0.6		

**Table 2:** SOC concentration at different soil depths of different forest types.

Soil depth (cm)	Average SOC content								Remarks
	Lower mixed hardwood (1000–1500 m)		Chir pine (1000–1600 m)		Upper mixed hardwood (1500–2700 m)		Oak (2300–2700 m)		
	Mean	SD	Mean	SD	Mean	SD	Mean	SD	Average
0–20	1.9	1.0	1.5	0.1	3.7	3.1	4.8	1.2	3.0
20–40	1.4	1.0	1	0.3	1.8	1.6	2.8	0.6	1.7
40–60	0.6	0.5	0.4	0.0	1.6	1.5	2.2	0.9	1.2

**Table 3:** Total SOC stock of different forest types at different soil depths.

Soil depth (cm)	SOC stock (t/ha)								Remarks
	Lower mixed hardwood		Chir pine		Upper mixed hardwood		Oak		
	Mean	SD	Mean	SD	Mean	SD	Mean	SD	Average
0–20	28.9	14.5	25.5	1.5	51.3	33.1	64.6	18.4	42.6
20–40	22.3	16.9	17.7	4.6	27.9	18.3	39.0	9.4	26.7
40–60	11.1	9.3	7.0	2.2	25.1	18.4	45.9	13.3	22.4
Total	62.5		50.8		104.4		149.6		

**Table 4:** ANOVA table for SOC stock of different forest types.

Source	Degree of freedom (df)	Sum of squares (SS)	Mean squares (MS)	*F*-stat	*p* value
Between groups	3	30.3	10.1	3.8	0.019
Within groups	32	84.4	2.6	—	—
Total	35	114.7	—	—	—

**Table 5:** ANOVA table for bulk density of different forest types.

Source	Degree of freedom (df)	Sum of squares (SS)	Mean squares (MS)	*F*-stat	*p* value
Between groups	3	0.15	0.05	4.1	0.014
Within groups	32	0.4	0.012	—	—
Total	35	0.56	—	—	—

**Table 6:** ANOVA table for SOC stocks in different forest types.

Source	Degree of freedom (df)	Sum of squares (SS)	Mean squares (MS)	*F*-stat	*p* value
Between groups	3	4484.7	1494.9	3.7	0.021
Within groups	32	12907.1	403.3	—	—
Total	35	17391.8	—	—	—

**Table 7:** ANOVA table of SOC stock between soil layers for Chir pine forest.

Source	Degree of freedom (df)	Sum of squares (SS)	Mean squares (MS)	*F*-stat	*p* value
Between groups	2	464.8	232.4	16.05	0.003
Within groups	6	86.8	14.4	—	—
Total	8	551.6	—	—	—

**Table 8:** ANOVA table of SOC stock between soil layers for lower mixed hardwood forest.

Source	Degree of freedom (df)	Sum of squares (SS)	Mean squares (MS)	*F*-stat	*p* value
Between groups	2	490.3	245.1	0.83	0.47
Within groups	6	1758.5	293.08	—	—
Total	8	2248.8	—	—	—

**Table 9:** ANOVA table of SOC stock between soil layers for upper mixed hardwood forest.

Source	Degree of freedom (df)	Sum of squares (SS)	Mean squares (MS)	*F*-stat	*p* value
Between groups	2	1155.3	577.6	0.6	0.55
Within groups	6	5330.6	888.4	—	—
Total	8	6485.9	—	—	—

**Table 10:** ANOVA table of SOC stock between soil layers for oak forest.

Source	Degree of freedom (df)	Sum of squares (SS)	Mean squares (MS)	*F*-stat	*p* value
Between groups	2	1745.6	872.8	2.8	0.13
Within groups	6	1882.03	313.6	—	—
Total	8	3627.6	—	—	—

## Data Availability

The data used to support the findings of this study are included within the article.
